# The occurrence and distribution of *Tuckerella japonica* (Acari: Tuckerellidae) on tea bushes, *Camellia sinensis* and *C. assamica,* in Alabama, Georgia and South Carolina, USA

**DOI:** 10.1007/s10493-016-0055-0

**Published:** 2016-06-13

**Authors:** Carl C. Childers, Timothy A. Ebert, Michael E. Rogers, Merle Shepard

**Affiliations:** Entomology and Nematology Department, Citrus Research and Education Center, University of Florida, IFAS, 700 Experiment Station Road, Lake Alfred, FL 33850 USA; 26 Wood Sorrel Lane, Hendersonville, NC 28792 USA; Coastal Research and Education Center, Clemson University, 2700 Savannah Hwy, Charleston, SC 29414 USA

**Keywords:** Tetranychoidea, Phytophagous mite, Theaceae

## Abstract

Adults, immatures and eggs of *Tuckerella japonica* (Ehara) were collected from unknown clones or varieties of *Camellia sinensis* (L.) O. Kuntze tea bushes in the Clemson University Farm, Coastal Research and Education Center, Charleston, South Carolina; from Assam hybrids in The Caw Caw Nature Preserve in Ravenel, SC; from *C. sinensis* and *C. assamica* (Masters) in the Charleston Tea Plantation on Wadmalaw Island, SC; *C. sinensis* in the Fairhope Tea Plantation in Fairhope, Alabama; and from *C. sinensis* ‘Rosea’ and a *C. sinensis* and *C. assamica* hybrid in Savannah and Ellabell, Georgia, between 1994 and 2015. This mite was consistently collected from 1-, 2- and 3+-year-old wood of tea plants with significantly greater numbers collected from 2-year-old wood. All stages of the mite were found within longitudinally split areas of the wood where underlying green bark tissues were exposed. As 1-year-old wood matured, there was increased splitting of the bark with increased mite presence. Mature green fruit (= developing seed pods) of tea were also frequented by *T. japonica* between June–July and October and their numbers were no greater than those on 1- or 3+-year wood. When the fruit were small (March–May) or as they hardened in late fall, they were not suitable feeding sites for this mite. Very few *T. japonica* were collected from 50 mature, inner or outer leaf samples with none usually found. *Tuckerella japonica* has multiple, overlapping generations and occurs on tea throughout the year in Alabama, Georgia and South Carolina, USA.

## Introduction

A fledgling tea industry is developing in the United States with Hawaii leading the way in number of tea farms (Zee et al. 2003; Hamasaki et al. 2008). At least 20 or more tea farms are located in Hawaii, Washington, Oregon, Texas, Mississippi, Alabama, Florida, South Carolina, Virginia and possibly one or two other states. Farms are in different stages of development ranging from potted cuttings of either known tea varieties or from mother plants of unknown origin to actual tea production (Anonymous 2014, Rosen 2014). With very few exceptions, the tea farms are quite small with the largest being the Charleston Tea Plantation on Wadmalaw Island in South Carolina with about 51 ha (Webster 2000).

Commercial tea cultivation in the United States was first attempted when tea seeds were sent to Savannah, Georgia in 1744 (Phillips 2007). Tea was later grown in Greenville, South Carolina from 1848 to 1853 and in Georgetown, South Carolina from 1874 to 1879 (Walcott 1999). Phillips (2007) and Beard et al. (2013) reviewed the history of tea and its introduction into mainland USA.

*Camellia sinensis* (L.) O. Kuntze, *C. assamica* (Masters), *C. assamica lasiocalyx* (Planch.MS) and *C. japonica* L. are all grown for tea production in different places worldwide (Tea Research Association 2015). Cuttings from mother plants are required for propagation as seed produced plants are not identical to the parent plant.

Initial contact with the tea plantation on Wadmalaw Island occurred in 1994 by the first author. The objective then was to look at the mite complex on tea for predators as many mite pests, especially eriophyoid species, were reported from most tea producing areas in the world (Rao 1974; Muraleedharan et al. 1988; Channabasavanna 1996; Hazarika et al. 2009). Also, pesticides were reportedly not used at this farm thus offering opportunities to find potential predators of eriophyoid mite pests on citrus. During preliminary sampling in 1994, *Tuckerella japonica* Ehara (Acari: Tuckerellidae) was found on tea bushes at the farm. The purpose of this paper is to report on the occurrence, geographical and within-plant distribution of *T. japonica* on clones of *C. sinensis* and *C. assamica* in different tea farms and dooryard settings in Alabama, Georgia and South Carolina, USA.

The Tuckerellidae is a small family of the Tetranychoidea with one genus and 28 species (Zhang 2011). According to Beard et al. (2013), there are four species of Tuckerellidae with broad geographical distributions including: *T. ornata* (Tucker) originally described from South Africa, *T. knorri* Baker & Tuttle described from Thailand and *T. pavoniformis* (Ewing) described from Hawaii. The fourth species, *T. japonica* Ehara, was described from Japan and occurs in Australia, New Zealand, China, Philippines, Vietnam, Italy and South Carolina, USA.

## Materials and methods

On 5 October 1994, three tea bushes at the Coastal Research and Education Center Farm, Clemson University in Charleston were sampled for mites. Three vertical divisions of each bush were sampled following the method of Muraleedharan et al. (1988). The divisions included taking lengths of a branch equally divided into distal (= upper), middle and basal levels from each of the three plants selected. Lateral shoots and leaves were included within each of the three levels for each branch sampled. Two branches from each of the three plants were cut using pruning shears into approximately equal lengths per division and each pair of branches was washed separately in a bucket containing approximately 250 ml of 80 % ethanol (Childers and Ueckermann 2015). The tea bushes differed in size at the farm so the sampled lengths of the three divisions of branch cuttings varied from 30.5 to 37.5 cm each. A fourth treatment consisted of 50 mature leaves from the upper part of the tea bush canopy. The four treatments were collected from each of the same tea bushes and replicated three times.

On 5 October 1994, Block 1 at the Charleston Tea Plantation was sampled for mites, including *T. japonica*. Treatments included: (1) 2 m of branches, associated lateral shoots and leaves from the distal third of the plant, (2) 2 m of branches, associated lateral shoots and leaves from the mid third of the plant, (3) 2 m of branches, associated lateral shoots and leaves from the basal third of the plant, and (4) 50 mature leaves collected from the distal area of each of the same plants sampled. The plants were selected at random and replicated six times.

Beginning in October 2013, the sampling method was changed to establish exactly where, when and in what comparative numbers that *T. japonica* occurred within tea bushes. Five different dates between 9 October 2013 and 27 October 2014 were sampled in Block 1 at the Charleston Tea Plantation and treatments included: (1) 50 inner canopy leaves, (2) 50 outer canopy leaves, (3) lengths of 1-year-old wood cuttings that totaled 244 cm, (4) lengths of 2-year-old wood cuttings that totaled 244 cm, (5) lengths of 3-year-old and older wood cuttings that totaled 244 cm, and (6) 25 fruit (= seed pods). Eight or more lengths of branches totaling 244 cm were cut at random for each of the three age classes of wood from different tea plants, measured to required lengths and immediately washed in the alcohol solution for each replicate. One additional treatment (7) 100 open flowers were collected at random in the 10 September 2013 test only. Each treatment was replicated six times and each sample replicate was washed separately in a bucket containing approximately 250 ml of 80 % ethanol. Each plant sample was vigorously agitated in solution and the plant materials discarded. The rinsate was poured into a labeled glass jar and returned to the laboratory for processing. Many of the mite species would rapidly leave a disturbed plant during sampling. Therefore, rapid preservation of the fauna was required to accurately measure what was present within each sample.

Data analysis was completed using generalized linear models (Gbur et al. 2012) implemented in Proc GLIMMIX in SAS/STAT software, v.9.4 of the SAS system (SAS Institute, Cary, NC, USA). Mean comparisons were completed using square root transformed data and the Tukey honestly significant difference (HSD) multiple range test. Although the test was completed on transformed data, the means are shown as untransformed values. Some sample dates and locations had insufficient sample size for statistical analysis and these results are presented as raw means.

A small understory planting of three or four *C. assamica* trees was sampled at the Charleston Tea Plantation on 27 October 2014. One wood sample with a length of 244 cm was collected from each of two trees with each measured from the tip of the branch downward. Age of the wood could not be determined. Two separate 50 mature leaf and two 25 fruit samples were collected separately from each of the same trees. Each sample was washed separately in alcohol as previously stated.

The Caw Caw Nature Preserve in Ravenel, SC was sampled on 28 October 2014. Three replicates of treatments consisting of (1) 244 cm of main branch cuttings, (2) 50 mature leaves and (3) 25 fruit were collected separately and at random then washed in 80 % ethanol as above. It was difficult to identify the ages of wood in this planting so lengths were determined from the newest growth at the tip of a branch downward.

The Fairhope Tea Plantation in Alabama was sampled on 29 October 2014. Three replicates of each of the following treatments: (1) 50 inner leaves, (2) 50 outer leaves, (3) 244 cm of 1-year-old wood cuttings, (4) 244 cm of 2-year-old wood cuttings, (5) 244 cm of 3-year-old or older wood cuttings, and (6) 25 fruit were collected separately at random and processed as above. This tea planting was sampled again on 26 September 2015 using 50 inner and 50 outer leaves and three ages of wood collected as above and replicated six times.

Multiple non-replicated samples of *C. sinensis* leaves and wood were collected from one tea planting in Pickens, South Carolina, in July 2015. Three locations in Mississippi were sampled: one near Brooksville in August 2014, another at Mississippi State University and one in Poplarville, both in September 2015. One dooryard tea planting each was sampled in New Orleans and in the Hammond, Louisiana vicinity in September 2015 and one small tea farm in Mount Vernon, Texas in October 2015. Wood, leaves and flowers of many *C. sinensis* varieties were sampled separately as above in Savannah and Ellabell, Georgia during 15–16 November 2015.

## Results and discussion

*Tuckerella japonica* motiles were collected from the distal, mid and basal areas of tea branches, lateral shoots and associated leaves at the Clemson University Farm on 5 October 1994, whereas none were found on the 50 leaf samples (Table 1). Further sampling was not repeated due to limited numbers of single planted tea bushes and existing variability among those plants. Possible pesticide drift issues from adjacent cultivated fields and potential mite mortality were also of concern.

**Table 1:** Mean (± SD) number of *Tuckerella japonica* motile stages collected from three vertical divisions of tea plants at the Clemson University, Coastal Research and Education Center, Charleston, SC, USA, on 5 October 1994

Treatment^a^	Number of mites
Distal	19.33 ± 27.59
Mid	23.50 ± 24.39
Basal	12.33 ± 3.69
50 mature leaves from the distal division	0 ± 0

^a^Treatments replicated three times

In the 10 September 2013 sampling at the Charleston Tea Plantation, *T. japonica* numbers were greater on the 2-year-old wood versus mature leaves, 3-year-old and older wood or 100 open flowers, whereas mite numbers on 1-year-old wood and 25 fruit were not significantly different (Table 2). Mite numbers on 2-year-old wood were significantly greater than the other treatments in four additional field experiments conducted between 20 February and 27 October 2014 at the Charleston Tea Plantation (Table 2). When the five dates of wood samples were combined, the mean numbers of *T. japonica* remained significantly different on 2-year-old wood (Table 3).

**Table 2:** Mean (± SD) number of *Tuckerella japonica* motile stages collected from different sample types at the Charleston Tea Plantation in South Carolina

Sample type	10 September 2013	20 February 2014	12 May 2014	7 July 2014	27 October 2014
50 inner leaves	–	0.17 ± 0.41c	0 ± 0d	0 ± 0c	0.17 ± 0.41d
50 outer leaves	0.5 ± 1.22c	0 ± 0c	0.17 ± 0.41d	0 ± 0c	0 ± 0d
1-year-old wood	30.0 ± 44.80ab	4.5 ± 1.87b	18.17 ± 7.33b	9.5 ± 8.38b	5.83 ± 4.88bc
2-year-old wood	88.17 ± 82.14a	42.17 ± 18.18a	30.83 ± 11.46a	48.17 ± 14.11a	55.83 ± 34.64a
3+-year-old wood	7.33 ± 4.84b	4.5 ± 6.86b	3.33 ± 2.50c	18.17 ± 13.42b	14.67 ± 6.19b
25 green fruit	35.83 ± 53.01ab	–	0 ± 0d	10.5 ± 8.24b	4.67 ± 4.93c
100 open flowers	0 ± 0c	–	–	–	–
F	23.05	40.4	44.81	23.79	33.28
	Pr > F < 0.0001	Pr > F < 0.0001	Pr > F < 0.0001	Pr > F < 0.0001	Pr > F < 0.0001

Means within a column followed by different letters are significantly different (Tukey HSD: *P* < 0.05)

Fruit were pea-sized and no mites were present on 20 February (Table 2). However, as the green fruit sized from July through October, tuckerellid mite numbers increased. By October, many fruit were turning black as they hardened. Mite numbers began to decline as the maturing seed pods dried. At some point during late fall or winter, the fruit dry and split with the seeds scattering to the plant bed below. Removal of the seed pods reduces the problem of undesirable, non-clonal plants germinating within the beds. However, there would be no benefit in removing these fruit to reduce *T. japonica* populations as proposed by Beard et al. (2013) as numbers are only a small fraction of the mite population that occurs on the wood, especially 2-year-old branches.

**Table 3:** The effect of wood age on abundance of *Tuckerella japonica* on *Camellia sinensis* with dates combined

Effect	Numerator DF	Denominator DF	F	Pr > F
Block	5	87	14.54	<0.0001
Treatment	2	87	55.48	<0.0001
Block * treatment	10	87	1.79	0.077

	LS mean	SEM		R^2^
1-year-old wood	11.0833b	4.2783		0.70
2-year-old wood	44.8611a	4.2783		
3+-year-old wood	6.7333b	4.2783		

Means followed by different letters are significantly different (Tukey HSD: *P* < 0.05)

*Tuckerella japonica* was collected from three age classes of wood samples taken from the Fairhope Tea Plantation on 29 October 2014 (Table 4). A second replicated sample was taken on 26 September at this location and the 1- and 2-year-old wood samples were not significantly different, whereas the mean numbers of *T. japonica* motiles on 2-year-old wood samples were significantly greater than those on the leaf or 3+-year-old wood (Table 5). Growth and longitudinal splitting of the bark on 1-year-old shoots was more extensive compared with previous samples at other locations.

**Table 4:** Mean (± SD) number of *Tuckerella japonica* motile stages collected from different sample types at the Fairhope Tea Plantation in Alabama on 29 October 2014

Treatment^a^	Number of mites
50 inner leaves	0
50 outer leaves	0
1-year-old wood^b^	1.67 ± 1.53
2-year-old wood^b^	15.0 ± 24.27
3+-year-old wood^b^	2.0 ± 2.0
25 fruit	0

^a^Treatments replicated three times

^b^Each replicate consisted of 244 cm of specific aged wood

**Table 5:** Mean (± SD) number of *Tuckerella japonica* motile stages collected from different sample types at the Fairhope Tea Plantation in Alabama on 26 September 2015

Sample type	Number of mites
50 inner leaves	0 ± 0b
50 outer leaves	0 ± 0b
1-year-old wood	3.0 ± 6.16ab
2-year-old wood	4.0 ± 2.0a
3+-year-old wood	0.4 ± 0.89b
25 green fruit	–
100 open flowers	–

F = 5.88, Pr > F < 0.0027

Means followed by different letters are significantly different (Tukey HSD: *P* < 0.05)

Populations of this mite tended to increase into the fall period, then decline over the winter months. Increased mortality due to environmental conditions as well as predation by a complex of arthropod species and/or disease are possible contributing factors during the winter months as well as natural mortality due to environmental factors. A report on the arthropod predator complex on tea plants will be published in a separate paper.

Three *T. japonica* were collected from one 50 mature leaf sample on 9 October 2013. Three other *T. japonica* were collected from 2400 inner and outer leaves between 3 March and 27 October during this study. A total of 33 *T. japonica* were collected from two 244 cm lengths of wood cuttings from *C. assamica* trees that were understory plants in a densely wooded area at the Charleston Tea Plantation on 27 October 2014. Thirty-six *T. japonica* were collected from two samples of 25 fruit, whereas no mites were found on two 50-mature-leaf samples from the same plants. A single *T. japonica* was collected from a 50-mature-leaf sample of Assam tea at the Caw Caw Nature Preserve on 28 October 2014. *Tuckerella japonica* was not collected from either the three replicates of wood or fruit sampled and bark splitting was rarely observed.

Leaves and flowers of the *Camellia* plant hybrids or varieties sampled were not suitable feeding sites for *T. japonica*. An occasional tuckerellid would be collected from a leaf sample, but it was likely that the mite was moving to new feeding sites on the wood.

**Fig. 1:**
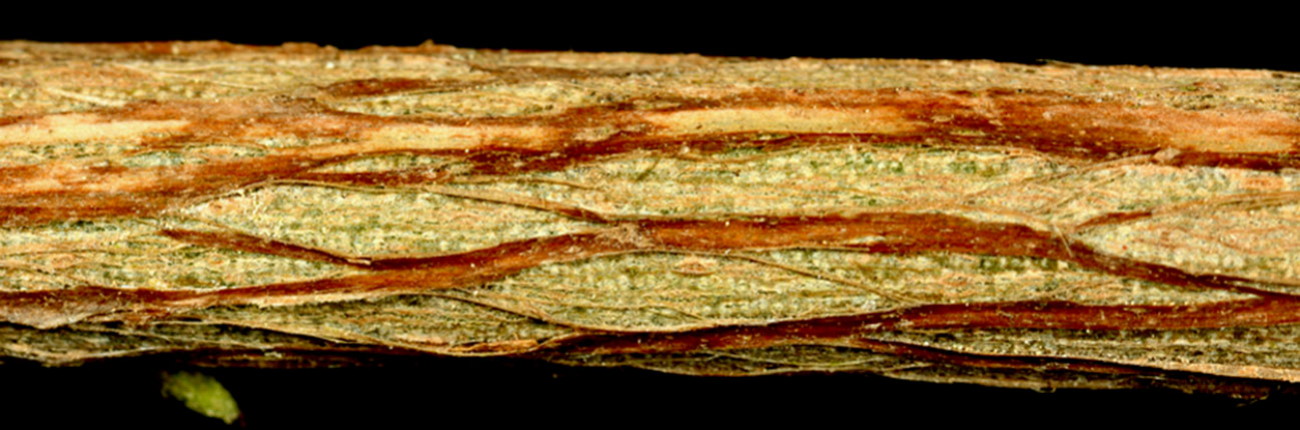
A 2-year-old branch from the Charleston Tea Plantation, Charleston, SC, USA. Note the longitudinal splitting and exposed green bark tissues

One-year-old wood on tea plants has much less splitting initially but increases with age as the season progresses. The extent of this splitting is far less evident on 1- versus 2-year-old wood. Hence, as the growing season progresses from March onward, new splitting becomes more evident on 1-year-old shoots and less so on the 2-year-old wood. At some point after 3 or 4 years, there is essentially no evident splitting of the outer bark as growth continues over time. The outer bark eventually forms a uniform covering on the older wood with little to no splitting evident. Motile stages and eggs of *T. japonica* are readily observed tucked under older bark that is peeled back from the surface of 2-year-old wood where *T. japonica* appears to be feeding (Fig. 1).

Three tea farms (one abandoned over a hundred years ago that is now part of the Caw Caw Nature Preserve in Ravenel, South Carolina, another that is being developed for tea production in Alabama and the Charleston Tea Plantation) all have populations of *T. japonica*. None of the four dooryard sites in Summerville, South Carolina yielded *T. japonica*; and, all three locations were near the original plantings of Charles Shepard (1893) at the Pinehurst Tea Plantation in Summerville. The tea plants at the Coastal Research and Education Center, Clemson University in Charleston also had *T. japonica*. Adults and immatures of *T. japonica* were collected on 1- and 2-year-old wood of the *C. sinensis* variety ‘Rosea’ and from a *C. sinensis*–*C. assamica* hybrid plant during 15–16 November 2015 in Savannah and from a ‘Rosea’ tea plant in Ellabell, Georgia. *Tuckerella japonica* was not found in Texas, Louisiana, or Mississippi during this study.

*Tuckerella japonica* larvae were collected from the Charleston Tea Plantation during May through November. Protonymphs were collected from February, April, June through September, November and December and deutonymphs from December through February and April through August. Tritonymphs were collected from February, June through September, November and December. Immatures were identified primarily by J.J. Beard from slide collections of the first author as well as those reported by Beard et al. (2013). Based on these data, *T. japonica* is present throughout the year on tea plants in the Charleston Tea Plantation with multiple overlapping generations.

The status of *Tuckerella* species as economic pests on tea or other crops remains uncertain. A report on feeding injury to bark splitting *C. sinensis* plants will be published in a separate paper. There has been much speculation of pest status and plant disease associations in several publications with no supportive data as noted by Zhang (2011). One of these reports was by Sudoi (1988) who stated that a species of *Tuckerella* was a pest on tea in Kenya. Validation of actual host plants for some of these species remains uncertain as no mention of tuckerellid eggs or immatures were noted along with the females used to describe new species (Zaher and Rasmy 1969; Ehara 1975).
